# Books Review

**Published:** 1869-12

**Authors:** 


### Books Review.

*The Physicians Hand-Book for* 1870. By WILLIAM ELMER, M.D., and ALBERT D. ELMER, M. D. New York: W. A. TOWNSEND & ADAMS, Publishers.

This is one of the most perfect and complete of its class, and comes to us in very tasty and elegant style. These Hand-Books are indispensable to the physician, and the only point is the selection. With the view of aiding our readers, we publish its table of contents: "Index to diseases, classification of diseases, ready method in asphyxia, poisons and their antidotes, diagnostic examination of urine, the pulse, list of incompatibles, medicinal weights and measures, abbreviations of medicinal properties of remedies, table of signs and abbreviations, materia medica, index of common names of remedial agents, art of writing prescriptions, record of practice, calendar for the year, names, addresses, bills and accounts, record of practice and treatment, tables of signs for daily practice, obstetric record, diagnostic record, obstetric calendar, general memoranda.

---

*Transactions of the Twenty-fourth Annual Meeting of the Ohio State Medical Society.* 1869.

The record of the Proceedings of this Society shows how deep an interest is felt in it by the profession of the State. The meeting was very largely attended, and the volume of Transactions contains articles from the most distinguished members of the profession in Ohio. The address of the retiring President, Dr. Alex. Dunlap; A. M., M. D., and Reports upon Intra-Cranial Tumors by Dr. Roberts Barthalo, Haematica, by Dr. E. H. Hyatt; Carcinoma Uteri, by Dr. A. B. Jones; Hypodermic Medication, by Dr. J. W. Weaver; Ophthalmology, by Dr. A. D. Williams; Fel Bovinum, by Dr. Isaac Kay; Chlorinated Anaesthetics, by Dr. J. B. Hough; and Report upon Obituaries, by Dr. E. B. Stephens, are all papers of value and interest.

---

*First Annual Announcement of the Kansas City College of Physicians and Surgeons.*

This institution, which is situated on the western border of the state of Missouri, commences operation this month, by the giving of what is called a preliminary course of lectures, while the first regular course will be given one year hence. The professorships are ten in number, two of which are yet to be occupied; the fees are *regular*. The college building is new and favorably situated in the city, while the general location of the institution is such as will accommodate students from a wide region.

---

*A Compend of the Materia Medica.* By JOHN C. RILEY, A. M., M. D. Philadelphia: J. B. LIPPENCOTT & Co. 1869.

The author only claims for this work, that it is a "Compend of the Materia Medica, and not designed to be a full exposition of the subject." It is an outline description of the subjects named, and is designed mainly to aid the student during lectures, or perhaps to aid the practitioner. It appears to us well adapted to the object in view, and very well suited to those who desire brief, concise and practical knowledge upon any of the articles of Materia Medica, or any of the subjects in therapeutics.

---

*The Principles of Naval Staff Rank, with its History in the United States Navy for over half a century.* By a Surgeon of the U. S. Navy. 1869.

This work appears very opportunely, since the profession and government are at present considering the question of Naval Rank. The work contains a full index, and is in every respect a very interesting and instructive history, containing also the principles upon which Rank is based. We are under many obligations for the favor of receiving it.

---

### *Luxations of the Hip and Shoulder Joints, and the Agents which oppose their Reduction.* By Moses Gunn, A. M., M. D.

The author, in a well written monograph upon this subject, establishes the following general rule:—

In *all* dislocations, place the limb in just *the position which characterized it at the moment of escape*, and the reduction will then be easily affected.

We further lay down the following special rules:—

In the luxation upon the dorsum ilii, the patient lying on his back, carry the limb across its fellow at a point corresponding with the union of the middle with the upper third, rotate inwards, and the pelvis being fixed by an assistant, the head may now be readily drawn into its place.

In the dislocation into the obturator foramen, when extension is being made in the usual way at the upper part of the thigh, the limb should be *abducted* instead of *adducted*, as universally directed; *abduction* conforms to the general rule laid down above, and relaxes the upper and untorn portion of the ligament.

In the forward dislocation upon the pubis, while extension and counter-extension is being made in the usual manner, the limb should be rotated externally; this relaxes the posterior and untorn portion of the ligament.

In the backward luxation into the sciatic notch, the limb should be carried across the opposite groin, and rotated internally, previous to any extension being made.

In the luxation of the humeral head into the axilla, the arm should be drawn upward by the side of the head, as directed in my first article.

In the forward dislocation upon the thorax, the arm should be rotated externally before extension is attempted.

In the luxation backwards upon the dorsum scapulæ, the arm should be rotated internally before extension is commenced.

---

### *Electricity as a means of Diagnosis. With a tabulated statement of 500 cases of Disease, treated mainly by the method of General Electrization.* By A. D. Rockwell, A. M., M. D.

The author says: "The foundation principles, on which Electrization can be made a means of diagnosis of disease, are simply these two:

*First.*—The fact that all the parts and organs are more or less sensitive to the electric current, and that this sensitiveness is modified by disease.

*Second.*—The fact that the contractile power of the muscle, uuder the influence of the electric current (electro muscular contractility,) is more or less modified by disease.

